# Increasing putative vector importance of *Trichophoromyia* phlebotomines (Diptera: Psychodidae)

**DOI:** 10.1590/0074-02760190284

**Published:** 2020-02-03

**Authors:** Thiago Vasconcelos dos Santos, Fernando Tobias Silveira

**Affiliations:** 1 Ministério da Saúde, Secretaria de Vigilância em Saúde, Instituto Evandro Chagas, Seção de Parasitologia, Ananindeua, PA, Brasil; 2 Universidade Federal do Pará, Núcleo de Medicina Tropical, Belém, PA, Brasil

**Keywords:** leishmaniasis, psychodidae, attention, vector

## Abstract

Despite some phlebotomines being well recognised as vectors of leishmaniasis agents, vector importance of those belonging to the genus *Trichophoromyia* has not been extensively studied. The present study provides evidence regarding the putative vector role played by some species of *Trichophoromyia* on leishmanine enzootics, based on literature reports and findings obtained from field experiments conducted in the ecotopes of Pará State, Brazil. The species *Th. ubiquitalis*, *Th. velascoi*, *Th. auraensis*, *Th. ininii* and *Th. brachipyga* possess minimal criteria to be included in the list of suspected leishmanine vectors. However, knowledge on man-biting behavior, substantiation of vector competence and determination of epidemiological implications are limited for all of the above mentioned species. Published studies together with present data draw attention to prioritize these phlebotomine species in entomological surveillance programs and studies on experimental susceptibility to *Leishmania* spp. infection.

Phlebotomines (Diptera: Psychodidae: Phlebotominae) are a group of insects of medical importance. Their blood-feeding females are proven vectors of protozoan leishmanine species, mainly from the *Leishmania* genus (Euglenozoa: Trypanosomatidae: Leishmaniinae), the causative agents of a group of neglected zoonotic diseases, known as the leishmaniases.^1^ ^,^ ^2^

In the New World, life cycle of leishmanine parasites is maintained by a complex mosaic of vector/reservoir interactions, resulting in a variety of ecological arrangements with worrisome consequences in clinical manifestations, namely - American visceral leishmaniasis (AVL) and American cutaneous leishmaniasis (ACL).^3^ ^,^ ^4^ In the Americas, a total of 541 species - 524 current and 17 fossil species - have been described, and they are grouped into supra-specific categories (genera, subgenera, groups, and series of species).^5^ ^,^ ^6^ Among these, almost more than 20 species are considered vectors of leishmaniasis in the New World. This relatively small number of vectors is the function of a number of ecological, physiological and biochemical factors inherent to both insects and parasites. These factors influence the close contact between insect/parasite binomial, survival of the parasites in the digestive tract of the insect, and their multiplication and transformations that result in the development of infective forms, influencing their transmission to the vertebrate host.^3^

*Trichophoromyia* was originally established as a subgenus of *Lutzomyia* França, 1924 by Barretto^7^ and later upgraded to genus level.^8^ Its type-species is *Th. ubiquitalis* (Mangabeira, 1942) (formerly *Phlebotomus ubiquitalis*). To date, 44 species of *Trichophoromyia* have been reported in the New World.^6^ Females of several *Trichophoromyia* species are markedly similar, and therefore, identification is usually based on male morphology. The geographical overlapping of the species usually complicates the diagnosis.^5^ ^,^ ^9^

For many years, an apparently null medical importance of *Trichophoromyia* may have contributed to this phlebotomine genus to be understudied. *Trichophoromyia* was considered to have limited geographical distribution, predominantly in the Amazon basin, with unknown human biting behavior, and circumstantial history of natural infection by flagellates; obtained only when sharing same ecotope of *Leishmania* vector/reservoir systems, thus, not likely playing an important role as vectors of leishmaniases agents to humans, limiting them to the zoological interest.^9^ However, since the 1990s, some species have been found to be closely associated with ACL *foci* and have started garnering attention. Currently, five species of *Trichophoromyia* have been identified naturally infected using microscopic analysis and/or have been found to be *Leishmania* DNA positive based on the results of polymerase chain reaction (PCR) analyses, which is the first essential criterion to be regarded as suspected vectors.^1^ Data available on these species are summarised in Table and further discussed in the context of ACL epidemiology. In addition, the present study also includes our recent findings on entomological studies conducted in locations from the Brazilian State of Pará, as the mineral province of Carajás (municipality of Canaã dos Carajás), the Belo Monte hydroelectric system (municipality of Vitória do Xingu), the Bosque Rodrigues Alves - Jardim Botânico da Amazônia and the Mosqueiro Island (municipality of Belém).

**TABLE:** *Trichophoromyia* phlebotomine species associated with *Leishmania* spp.

*Trichophoromyia* species	Diagnosis method	*Leishmania* species	Reference
*Th. ubiquitalis*	Microscopically + MLEE Microscopically + MLEE Microscopically + RNA POLIILS RLFP-PCR ITS1 PCR + sequencing kDNA PCR + *hsp*70 PCR RFLP/sequencing ITS1 PCR + sequencing kDNA PCR	*L.* (*V.*) *lainsoni* *L.* (*V.*) *lainsoni* *L.* (*V.*) *lainsoni* *L.* (*Viannia*) sp.; *L.* (*L.*) *amazonensis* *L.* (*V.*) *lainsoni* *L.* (*V.*) *lainsoni* *Leishmania* sp.	Silveira et al.^(11)^ Lainson et al.^(12)^ Present work Silva et al.^(14)^ Pereira Junior et al.^(13)^ Quiroga et al.^(15)^ Resadore et al.^(16)^
*Th. velascoi*	Microscopically	n.i.	Martinez et al.^(19)^
*Th. auraensis*	kDNA PCR + FRET-based real time-PCR kDNA PCR kDNA-PCR + *hsp*70 sequencing kDNA PCR	*L.* (*V.*) *lainsoni* *Leishmania*. sp. *L.* (*V.*) *braziliensis* *Leishmania* sp.	Valdivia et al.^(20)^ Ogawa et al.^(23)^ Araújo-Pereira et al.^(25)^ Resadore et al.^(26)^
*Th. auraensis/Th. ruifreitasi*	PCR-based	*L.* (*V.*) *braziliensis*; *L.* (*V.*) *guyanensis*	Teles et al.^(27)^
*Th. ininii*	SSU rRNA qPCR + RFLP PCR microscopically kDNA-PCR + *hsp*70 sequencing	*Leishmania* sp. *Leishmania*. sp. *L.* (*V.*) *braziliensis*	Kent et al.^(30)^ Fouque et al.^(29)^ Vasconcelos dos Santos et al.^(31)^
*Th. brachipyga*	Microscopically + RNA POLIILS RLFP-PCR Microscopically + RNA POLIILS RLFP-PCR	*L.* (*V.*) *lainsoni* Microscopically + RNA POLIILS RLFP-PCR	Sánchez Uzcátegui et al.^(33)^ Present work
*Th. brachipyga*/*Th. adelsonsouzai*	Microscopically	n.i.	Present work

FRET-based: fluorescence resonance energy transfer-based; *hsp* 70: 70 kilodalton heat shock protein; ITS1: internal transcribed spacer 1; kDNA: kinetoplast DNA; MLEE: multilocus enzyme electrophoresis; n.i. not identified; PCR: polymerase chain reaction; RFLP: restriction fragment length polymorphism; RNA POLIILS: RNA polymerase II largest subunit; SSU rRNA qPCR: small-subunit ribosomal RNA.

***Trichophoromyia* species with putative vector importance**

*Trichophoromyia ubiquitalis* - In 1983, during entomological investigations of the river Paranapanema region, in the foothills of the Serra dos Carajás (Pará State, Brazil), Lainson, Shaw and Ready found a specimen of *Th. ubiquitalis* naturally infected, and the *Leishmania* isolate was designed, at that time, as an unnamed parasite of the subgenus *Viannia*.^10^ Years later, after re-examination, the isolate was recognized as *L.* (*Viannia*) *lainsoni* Silveira, Shaw, Braga & Ishikawa, 1987.

Silveira et al.^11^ performed entomological studies in the forested outskirts of Belém in Pará State, Brazil (recognised as the type locality of *L.* (*V.*) *lainsoni*, and the presumed infection site of ACL patients) and in the ecologically related forest of Utinga in Belém metropolitan region. Out of the 375 *Th. ubiquitalis* specimens captured, they found nine females to be highly infected with flagellates, and eight of these parasites were identified to be *L.* (*V.*) *lainsoni*. Under laboratory conditions, 71/83 specimens successfully bit humans, 48 h after capture. However, under field conditions, they did not find *Th. ubiquitalis* to be anthropophilic. Lainson et al.^12^ provided further observations on the laboratory and field behaviors of *Th. ubiquitalis* captured from the mineral province of Carajás, Pará State, Brazil. In their study, only one specimen attempted to bite a professional during field capture. In order to establish a colony, 64/80 flies previously confined in a nylon cage were fed on a volunteer at a time varying from 24-48 h after capture; strengthening the hypothesis that *Th. ubiquitalis* can be triggered to actively attack humans under unusual conditions. In an agricultural settlement of Mosqueiro Island in the outskirts of Belém, we recently investigated an ACL presumed infection site where *L.* (*V*.) *lainsoni* has been isolated from the cutaneous lesion of a dweller. Light traps installed in the intradomiciliary environment captured several *Th. ubiquitalis*, providing evidence for the potential indoor behavior of this species.

Other ecotopes distinct from those of the State of Pará, have also been recorded with *L.* (*V.*) *lainsoni/Th. ubiquitalis* association. During an entomological survey in Tefé municipality of Amazonas State, Brazil, Pereira Junior et al.^13^ found 7/60 pooled *Th. ubiquitalis*, captured in dry land forest environment, to be DNA positive for the 70 kilodalton heat shock protein (*hsp* 70) target, with sequences of *L.* (*V.*) *lainsoni*. In contrast, Silva et al.^14^ found *Th. ubiquitalis* DNA positive for *L. (L.) amazonensis* and *L. (Viannia)* sp*.* Quiroga et al.^15^ found *L.* (*V*.) *lainsoni* DNA in two peridomiciliary pooled samples of nine specimens of *Th. ubiquitalis* from Macas, Morona Santiago province, Ecuador, during the rainy season. In Porto Velho, Rondônia State, Brazil, two pools of *Th. ubiquitalis* tested positive for *Leishmania* sp. DNA, suggesting the putative vector role of this species in that municipality.^16^

Currently, there are strong evidences on the putative vector role of *Th. ubiquitalis* with respect to *L.* (*V.*) *lainsoni* transmission from distinct ecoregions of Brazil and Ecuador. In addition, spatiotemporal congruence of this phlebotomine species and ACL cases due to this parasite in other regions/countries, even with no findings of natural infection during entomological surveys, consubstantiate its vector candidacy. Intriguing findings about the southward expansion of *L.* (*V.*) *lainsoni* were recently reported in Paraguay;^17^ however, raise concerns on the existence of alternative vectors for this parasite, distinct from *Th. ubiquitalis* or other *Trichophoromyia* species*.* Furthermore, it is noteworthy to mention that *Pintomyia nuneztovari* (Ortiz, 1954) has already been found with *L.* (*V.*) *lainsoni* DNA in an ACL *focus* from the sub Andean region of Bolivia.^18^

*Trichophoromyia velascoi (Le Pont & Desjeux, 1992)* - During investigation on the first ACL case caused by *L.* (*V.*) *lainsoni* in Bolivia, several females of *Th. velascoi* were found to be infected naturally at the end of the rainy season (May) in Caranavi province; however, the parasites were not isolated at that time.^19^ Nonetheless, based on the spatiotemporal congruence of human/phlebotomine infections, the authors highlighted the need of further studies on putative vector importance of *Th. velascoi* in the context of ACL transmission in that region.

*Trichophoromyia auraensis (Mangabeira, 1942) and/or Th. ruifreitasi Oliveira, Teles, Medeiros, Camargo & Pessoa, 2015* - Using a fluorescence resonance energy transfer-based real-time polymerase chain reaction, Valdivia et al.^20^ found four *Trichophoromyia auraensis* pools with DNA of *L.* (*V*.) *lainsoni* and *Leishmania* (*V*.) *braziliensis* in Madre de Dios, Peru, with a minimum detection rate estimated to be 0.6%. Moreover, based on the studies conducted in Puno, Peru, anthropophilic behavior has been observed in *Th. auraensis*,^21^ although early hypothesis on misidentification of the particular *Trichophoromyia* taxon have been raised for this Peruvian department and reported elsewhere.^22^

Ogawa et al.^23^ detected *Leishmania* sp. DNA within three pools of *Th. auraensis* captured from caves in Rondônia State, Brazil, using kinetoplast DNA (kDNA) PCR. However, none of the samples were positive in the *hsp70* region for sequencing. Furthermore, Teles et al.^24^ published an epidemiological study conducted on approximately seven thousand phlebotomines collected from Assis Brasil in Acre State, Brazil. The female phlebotomines were grouped by pools and examined for the DNA tracks of *Leishmania*. Among the positive samples, specimens of *Th. auraensis/Th. ruifreitasi* (ambiguously identified females) contained the DNA tracks of *Leishmania* from the *L.* (*V.*) *braziliensis* complex at high concentrations, with a minimal detection rate of 2.05%. It was regarded as the first report on the infection of *Th. auraensis*/*Th. ruifreitasi* by *L.* (*V.*) *guyanensis* and *L.* (*V.*) *braziliensis* in Brazil. Interestingly, Araújo-Pereira et al.^25^ found 9/96 individually processed and unambiguously identified *Th. auraensis* positive to *Leishmania* kDNA, with three of them revealed as *L.* (*V.*) *braziliensis*. Resadore et al.^26^ found *Th. auraensis* DNA positive for *Leishmania* sp. (minimal detection rate of 8.33%) in Rondônia State, Brazil. Consistent with the complementary roles described in these previous studies, there is no doubt to regard *Th. auraensis* as a putative vector.^27^

In an ACL endemic area of Rio Branco, Acre State, where *Th. auraensis* was the numerically dominant *Trichophoromyia* species, engorged females of this genus were found positive for *Gallus gallus* blood through *cytochrome b* PCR analysis. Although birds do not play a direct role in the ACL epidemiology, their presence may influence on the population dynamics of phlebotomines with increasing population density in the peridomestic environment, which could contribute to increase the risk of the transmission of *Leishmania* to humans.^28^

*Trichophoromyia ininii Floch & Abonnenc, 1943* - During a study conducted to identify changing patterns in ACL transmission in French Guiana, Fouque et al.^29^ found *Th. ininii* as the most common species among the captured phlebotomines, where one specimen from Montsinery and one from Cacao were positive for flagellates under microscopic observation. Molecular typing indicated *Leishmania* genus, but the species was not identified. In order to broaden the knowledge on vectors involved in ACL transmission in Suriname, Kent et al.^30^ isolated a pooled sample of *Th. ininii* from Sabajo hills as *Leishmania* DNA positive. They were unable to identify the parasites due to insufficient amount of DNA. Studying an environmentally impacted area of Oiapoque outskirts, Amapá State, Brazil, we recently detected, within the phlebotomine composition of the forest border of an anthropized environment, two pooled samples of ten *Th. ininii* specimens were found to be DNA positive for *Leishmania* (*Viannia*) sp.; one sample allowed characterisation at species level, with sequence compatible with *L.* (*V*.) *braziliensis*.^31^

*Trichophoromyia brachipyga Mangabeira, 1942 and/or Th. adelsonsouzai Vasconcelos dos Santos, Silva, Barata, Andrade & Galati, 2013* - In the Belo Monte hydroelectric system affected area in Pará State, Brazil, a program monitoring ACL transmission has been implemented since 2012, providing substantial knowledge on ecology and taxonomy of phlebotomines. In addition to the findings, it rendered description of *Th. adelsonsouzai* based on males and females captured in Vitória do Xingu municipality.^32^ Interestingly, two females of *Trichophoromyia* were found infected with suprapylarian *Leishmania*-like promastigotes. Although *Th. adelsonsouzai* has been the dominant species in the infection site, representing more than 60% of phlebotomines captured on light traps, the coexistence of few males of *Th. brachipyga* has also been noted*.* Due the ambiguous taxonomic determination of closely related females of *Th. adelsonsouzai*/*Th. brachipyga*, and failure of *Leishmania* species characterisation, this putative ACL life cycle remains unclear.

Sánchez Uzcátegui et al.^33^ investigated phebotomines from an urban park of Belém, the Bosque Rodrigues Alves - Jardim Botânico da Amazônia, where one specimen of *Th. brachipyga* was found to be moderately/weakly (++) parasitised with a peripylarian flagelate. After successful *in vitro* isolation, the restriction fragment length polymorphism - PCR (RFLP - PCR) characterisation showed a profile identical to that of *L.* (*V.*) *lainsoni* reference strain. Although in that site, *Th. ubiquitalis* was also present and expected to be infected by *L.* (*V.*) *lainsoni*, been recorded as the second most frequent species (14%), followed by *Th. brachipyga* (10.4%), no infection was diagnosed in this phlebotomine through microscopic examination. This was the first report of *Th. brachipyga* naturally infected by *L.* (*V.*) *lainsoni*.

We continued conducting studies in the mineral province of Carajás in Pará State, Brazil, and captured phlebotomines using light traps in a forest environment of Canaã dos Carajás municipality. In the field campaign of April 2019, we identified four *Th. ubiquitalis* and two *Th. brachipyga* naturally infected with moderate to luxuriant-growing promastigotes. The gut contents were inoculated into culture media and hind-feet of golden hamsters (ethical approval under protocol CEUA/IEC/SVS/MS n.10/2014). All strains were successfully isolated from one of these two described methods and thereafter characterised by PCR-RFLP technique. Digestion profiles of *Leishmania* strains from both phlebotomine species were found to be similar and compatible with *L.* (*V.*) *lainsoni*. By including observations made by Lainson et al.^12^ for Carajás, the vector role of *Th. ubiquitalis* with respect to *L.* (*V*.) *lainsoni* is thus, well established. These novel findings for *Th. brachipyga* corroborate with the results reported by Sánchez Uzcátegui et al.^33^ It is speculated that this *Trichophoromyia* species may act as an alternative vector, probably sharing its role with *Th. ubiquitalis* in some ecotopes.

*Concluding remarks* - The present study assembled data suggesting the possibility of occurrence of leishmanine emerging transmission patterns, where some species of *Trichohoromyia* may act as putative vectors of ACL agents, and probably also share roles with each other, while co-habiting the same ecotope. In nature, *Leishmania* species may have enzootic and zoonotic cycles as well, that may geographically overlap, maintained by different, but closely related phlebotomine species. As stated by Ready,^34^ putative vectors in previously unexplored *foci* can be targeted simply by their close taxonomic relationship with known vectors, using presumed evolutionary relationships in which *taxa* of parasites and vectors are linked together by unique behavioral or molecular phenotypes of epidemiological importance. As *Nyssomyia* and *Psychodopygus*, the most medically important phlebotomine genera for the New World, concentrate several vector species of *Leishmania*, *Trichophoromyia* may also include species possessing common conditions suitable for parasite development. Circumstantial ingestion of parasites for some of these species, however, is also likely. In this regard, long-term surveys are required to strength evidences on the ability of *Trichophoromyia* to sustain a natural cycle in sites where these apparently occasional infections have been recorded.

The number of new *Trichophoromyia* species described has been increased in the last years.^6^ Most of these descriptions were provided from specimens collected in environmentally impacted ecotopes of the Amazon Basin, where ACL is considered a prioritised disease to be monitored in vector surveillance programs. Numerical dominance in light-trap captures, permissiveness for *Leishmania* infection and spatiotemporal congruence with ACL epidemiology are usually variables considered for drawing attention to the possible involvement of *Trichophoromyia* in ACL enzootics.

The raising question on the putative vector importance of *Trichophoromyia* is also likely to be biased toward the marked increase in the general list of suspected vectors of leishmaniasis in the New World,^1^ ^,^ ^3^ ^,^ ^4^ which has been supported in the last years, mainly due to the results of PCR-based techniques for diagnosing *Leishmania* DNA targets within pooled or individually processed phlebotomines. Integrative molecular methodological approaches let us to include suspected vector species from phlebotomine genera with little or even null association with *Leishmania*, such as *Pintomyia*,^35^ *Pressatia*, *Evandromyia*,^25^ ^,^ ^31^ *Martinsmyia*, and *Micropygomyia*;^36^ in our view, care must be taken on interpreting these intriguing results. Even considering established natural infection, quantification of the parasitic load and/or demonstration of infective forms would certainly present a further step toward improving knowledge on putative *Leishmania* vectors inferred exclusively through DNA-based findings.

In fact, records of natural *Leishmania* spp. infections in *Trichophoromyia* phlebotomines are increasing but these facts, alone, do not necessarily correlates with an epidemiological or medical issue on leishmaniases. Low man-biting behavior of *Trichophoromyia* may contribute to the sporadically ACL due to their related *Leishmania* species, as occur with the epidemiology of *L.* (*V*.) *lainsoni*. Furthermore, studies on the knowledge of blood-feeding behavior, including attraction to bit man and *Leishmania* reservoir hosts, ecological association with ACL determinants, experimental susceptibility for *Leishmania* infection/ transmission, and mathematical modeling of vector/disease dynamics, must be improved to better comprehend vector-parasite interactions and their transmission cycles in ecotopes where these *Trichophoromyia* species are supposed to be transmitting *Leishmania* parasites.

## References

[1] Ready PD (2013). Biology of phlebotomine sandflies as vectors of disease
agents. Annu Rev Entomol.

[2] Espinosa OA, Serrano MG, Camargo EP, Teixeira MMG, Shaw JJ (2018). An appraisal of the taxonomy and nomenclature of trypanosomatids
presently classified as Leishmania and Endotrypanum. Parasitology.

[3] Brazil RP, Rodrigues AAF, Andrade JD (2015). Sand fly vectors of Leishmania in the Americas - A mini
Review. Entomol Ornithol Herpetol.

[4] Rangel EF, Lainson R, Costa SM, Shaw JJ, Carvalho BM, EF Rangel, JJ Shaw (2018). Sand fly vectors of American cutaneous leishmaniasis in
Brazil. Brazilian sand flies: biology, taxonomy, medical importance and
control.

[5] Galati EAB, EF Rangel, JJ Shaw (2018). Phlebotominae (Diptera, Psychodidae): classification, morphology,
and terminology of adults and identification of American
taxa. Brazilian sand flies: biology, taxonomy, medical importance and
control.

[6] Vasconcelos dos Santos T.Santos Neto NF.Sánchez Uzcátegui
YDV.Galardo AKR (2019). Trichophoromyia iorlandobaratai (Diptera Psychodidae), a new
phlebotomine species from the Brazilian Amazonia. J Med Entomol.

[7] Barretto MP (1962). Novos subgêneros de Lutzomyia França, 1924 (Psychodidae,
subfamília Phlebotominae). Rev Inst Med Trop São Paulo.

[8] Galati EAB, EF Rangel, R Lainson, orgs (2003). Morfologia, terminologia de adultos e identificação dos táxons da
América. Flebotomíneos do Brasil.

[9] Young DG, Duncan MA (1994). Guide to the identification and geographic distribution of
Lutzomyia sand flies in Mexico, the West Indies, Central and South America
(Diptera: Psychodidae). Mem Am Entomol Inst.

[10] Lainson R, Shaw JJ (1987). Evolution, classification and geographical distribution. In: W
Peters, R Killick-Kendrick, editors. The leishmaniases in biology and
medicine. Vol. 1. Biology and Epidemiology. Academic Press.

[11] Silveira FT, Souza AAA, Lainson R, Shaw JJ, Braga RR, Ishikawa EEA (1991). Cutaneous leishmaniasis in the Amazon Region natural infection of
the sandfly Lutzomyia ubiquitalis (Psychodidae: Phlebotominae) by Leishmania
(Viannia) lainsoni in Pará State, Brazil. Mem Inst Oswaldo Cruz.

[12] Lainson R, Shaw JJ, Souza AAA, Silveira FT, Falqueto A (1992). Further observations on Lutzomyia ubiquitalis (Psychodidae
Phlebotominae), the sandfly vector of Leishmania (Viannia)
lainsoni. Mem Inst Oswaldo Cruz.

[13] Pereira AM, Teles CB, dos Santos APA, Rodrigues MS, Marialva EF, Pessoa FA, Medeiros JF (2015). Ecological aspects and molecular detection of Leishmania DNA Ross
(Kinetoplastida Trypanosomatidae) in phlebotomine sandflies (Diptera:
Psychodidae) in terra firme and várzea environments in the Middle Solimões
Region, Amazonas State, Brazil. Parasit Vectors.

[14] Silva TRR, Assis MDG, Freire MP, Rego FD, Gontijo CMF, Shimabukuro PHF (2014). Molecular detection of Leishmania in sand flies (Diptera
Psychodidae: Phlebotominae) collected in the Caititu Indigenous Reserve of
the municipality of Lábrea, State of Amazonas, Brazil. J Med Entomol.

[15] Quiroga C, Cevallos V, Morales D, Baldeón ME, Cárdenas P, Rojas-Silva P (2017). Molecular identification of Leishmania spp in sand flies
(Diptera: Psychodidae, Phlebotominae) from Ecuador. J Med Entomol.

[16] Resadore F, Pereira AM, de Paulo PFM, Gil LHS, Rodrigues MMS, Araújo MDS (2019). Composition and vertical stratification of phlebotomine sand fly
fauna and the molecular detection of Leishmania in forested areas in
Rondônia State municipalities, western Amazon, Brazil. Vector Borne Zoonotic Dis.

[17] Benítez IR, Andrade-Zampieri R, Silveira-Elkhoury AN, FLoeter-Winter LM, Lauletta-Lindoso JA, Pereira J (2019). Detección molecular de Leishmania (Viannia) lainsoni Silveira,
Shaw, Braga & Ishikawa, 1987 (Kinetoplastida Trypanosomatidae) en
humanos de Paraguay. Rev Inst Med Trop.

[18] Bastrenta B, Buitrago R, Vargas F, Le Pont F, Torrez M, Flores M (2002). First evidence of transmission of Leishmania (Viannia) lainsoni
in a Sub Andean region of Bolivia. Acta Trop.

[19] Martinez E, Le Pont F, Mollinedo S, Cupolillo E (2001). A first case of cutaneous leishmaniasis due to Leishmania
(Viannia) lainsoni in Bolivia. Trans R Soc Trop Med Hyg.

[20] Valdivia HO, de los Santos MB.Fernandez R.Baldeviano GC.Zorrilla VO.Vera H.et
l (2012). Natural Leishmania infection of Lutzomyia (Trichophoromyia)
auraensis in Madre de Dios, Peru, detected by a fluorescence resonance
energy transfer-based real-time polymerase chain reaction. Am J Trop Med Hyg.

[21] Arístides H (1999). La leishmaniasis tegumentaria en el Alto Tambopata, Departamento
de Puno, Peru. Rev Med Exp.

[22] Fernandez R, Galati EB, Carbajal F, Wooster MT, Watts DM (1998). Notes on the phlebotomine sand flies from the Peruvian
southeast-I Description of Lutzomyia (Helcocyrtomyia) adamsi n. sp.
(Diptera: Psychodidae). Mem Inst Oswaldo Cruz.

[23] Ogawa GM, Pereira AM, Resadore F, Ferreira RGM, Medeiros JF, Camargo LMA (2016). Sandfly fauna (Diptera Psychodidae) from caves in the State of
Rondônia, Brazil. Braz. J Vet Parasitol.

[24] Teles CBG, dos Santos APA, Freitas RA, de Oliveira AFJ, Ogawa GM, Rodrigues MS (2016). Phlebotomine sandfly (Diptera Psychodidae) diversity and their
Leishmania DNA in a hot spot of American Cutaneous Leishmaniasis human cases
along the Brazilian border with Peru and Bolivia. Mem Inst Oswaldo Cruz.

[25] Araujo-Pereira T, de Pita-Pereira D, Boité MC, Melo M, da Costa-Rego TA, Fuzari AA (2017). First description of Leishmania (Viannia) infection in
Evandromyia saulensis, Pressatia sp and Trichophoromyia auraensis
(Psychodidae: Phlebotominae) in a transmission area of cutaneous
leishmaniasis in Acre State, Amazon Basin, Brazil. Mem Inst Oswaldo Cruz.

[26] Resadore F, Pereira AM, Carvalho LPC, dos Santos APA, Teles CBG, Medeiros JF (2017). Phlebotomine sand fly composition (Diptera Psychodidae) and
putative vectors of American cutaneous leishmaniasis in Porto Velho
municipality, western Amazon, Brazil. J Med Entomol.

[27] Teles CBG, Pessoa FAC, Medeiros JF, Camargo LMA, Araujo-Pereira T, Pita-Pereira D (2017). Trichophoromyia auraensis is a putative vector. Mem Inst Oswaldo Cruz.

[28] Ávila MM, Brilhante AF, Souza CF, Bevilacqua PD, Galati EAB, Brazil RP (2018). Ecology, feeding and natural infection by Leishmania spp of
phlebotomine sand flies in an area of high incidence of American tegumentary
leishmaniasis in the municipality of Rio Branco, Acre,
Brazil. Parasit Vectors.

[29] Fouque F, Gaborit P, Issaly J, Carinci R, Gantier J-C, Ravel C (2007). Phlebotomine sand flies (Diptera Psychodidae) associated with
changing patterns in the transmission of the human cutaneous leishmaniasis
in French Guiana. Mem Inst Oswaldo Cruz.

[30] Kent A, Vasconcelos dos Santos T.Gandadin A.Samjhawan A.Mans DRA.Schallig
HDFH (2013). Studies on the sand fly fauna (Diptera Psychodidae) in
high-transmission areas of cutaneous leishmaniasis in the Republic of
Suriname. Parasit Vectors.

[31] Vasconcelos dos Santos T.de Pita-Pereira D.Araujo-Pereira T.Britto
C.Silveira FT.Povoa MM (2019). Leishmania DNA detection and species characterization within
phlebotomines (Diptera Psychodidae) from a peridomicile-forest gradient in
an Amazonian/Guianan bordering area. PLoS One.

[32] Vasconcelos dos Santos T.da Silva FMM.Barata IR.de Andrade AJ.Galati
EAB (2014). A new species of phlebotomine, Trichophoromyia adelsonsouzai
(Diptera Psychodidae) of Brazilian Amazonia. Mem Inst Oswaldo Cruz.

[33] Sánchez Uzcátegui YDV, Vasconcelos dos Santos T, Silveira FT, Ramos PKS, dos Santos EJM, Póvoa MM (2019). Phlebotomines (Diptera: Psychodidae) from a urban park of Belém,
Pará State, northern Brazil and potential implications in the transmission
of American cutaneous leishmaniasis. J Med Entomol.

[34] Ready PD (2000). Sand fly evolution and its relationship to Leishmania
transmission. Mem Inst Oswaldo Cruz.

[35] Pita-Pereira D, Souza GD, Araujo-Pereira T, Zwetsch A, Britto C, Rangel EF (2011). Lutzomyia (Pintomyia) fischeri (Diptera Psychodidae:
Phlebotominae), a probable vector of American cutaneous leishmaniasis:
detection of natural infection by Leishmania (Viannia) DNA in specimens from
the municipality of Porto Alegre (RS), Brazil, using multiplex PCR
assay. Acta Trop.

[36] Rêgo FD, Rugani JMN, Shimabukuro PHF, Tonelli GB, Quaresma PF, Gontijo CMF (2015). Molecular detection of Leishmania in phlebotomine sand flies
(Diptera Psychodidae) from a cutaneous leishmaniasis focus at Xakriabá
Indigenous Reserve, Brazil. PLoS One.

